# RESILSTIGMA. Resilience to self-stigmatization experienced by people living with HIV: Which self-reported factors improve awareness among health workers?

**DOI:** 10.1371/journal.pone.0311776

**Published:** 2025-02-06

**Authors:** Christine Jacomet, Cécile Miele, Emilie Goncalves, Céline Lambert, Clément Belletier, Françoise Linard, Josiane Phalip-Le Besnerais, Pierre Dellamonica, Michaël Dambrun

**Affiliations:** 1 Service d’infectiologie, CHU Clermont-Ferrand, Clermont-Ferrand, France; 2 Laboratoire QualiPsy EE 1901, Université de Tours, Tours, France; 3 Unité de Biostatistiques, DRCI, CHU Clermont-Ferrand, Clermont-Ferrand, France; 4 Laboratoire de Psychologie Sociale et Cognitive (LAPSCO, UMR 6024), Université Clermont Auvergne, Clermont-Ferrand, France; 5 Service d’infectiologie, CHU Tenon-APHP et Hôtel Dieu APHP Paris, Paris, France; 6 Service d’infectiologie, CH Saint-Denis, Saint-Denis, France; 7 Université côté d’Azur, Nice, France; Stellenbosch University, SOUTH AFRICA

## Abstract

**Background:**

Self-stigmatization is the process by which environmental stigmas are internalized. It leads to a decline in self-esteem, isolation, denial and risk behavior, and impairs quality of life. The aims of our study were to investigate the psychological, social and medical factors, in particular psychological flexibility and its defusion component, that are associated with resilience to self-stigmatization in people living with HIV (PLHIV), and to assess clinicians’ perceptions of the condition.

**Methods:**

A multicenter observational study was conducted in France using self-reports from PLHIV consulted between January 15, 2022 and June 15, 2022 and from professionals responsible for their care (study registration number 2021-A01588-33/SI:21.02814.000036). Self-stigmatization was measured by examining four domains: perceived stigma, anticipated stigma, internalized stigma and enacted stigma.

**Results:**

Self-reports were collected from 45 hospital wards, 666 from PLHIV, of whom 71% were male with a mean age of 53 +/- 12.6 years, and 131 from health professionals, of whom 72.5% were clinicians. A total of 279 PLHIV (42%) reported items of self-stigmatization. Multivariable analysis showed that self-stigmatization was significantly associated with major depression (OR: 3.59, 95% CI: 1.19 to 10.80, p = 0.02), psychological inflexibility (OR: 1.53, 95% CI: 1.19 to 1.97, p = 0.001), and parental support deficit in childhood (OR parental support: 0.63, 95% CI: 0.40 to 0.98, p = 0.04). Among the dimensions of psychological inflexibility associated with HIV self-stigma, only fusion was positively associated (p<0.01). The proportion of PLHIV experiencing self-stigma was accurately estimated by only 31 (23.7%) health workers. Those health workers who did not minimize the prevalence of self-stigmatization among PLHIV had no particular characteristics.

**Conclusions:**

While the best means to combat self-stigmatization would be a social-ecological approach, it is fundamental to target in parallel individual vulnerability and protective factors accessible to health workers’ interventions. Psychiatric care and/or the new cognitive-behavioral therapies could be offered more often as part of personalized care.

## Introduction

Ending the HIV epidemic by 2030 will require tackling stigma more systematically and on a larger scale than current efforts [1]. Global evidence shows that stigma, poverty and discrimination are barriers to achieving each of the 95-95-95 targets: 95% of people living with HIV (PLHIV) tested, 95% on treatment, and 95% with an undetectable viral load [2]. They also undermine HIV testing, linkage to care, treatment adherence and viral load suppression [3]. According to the latest national evaluation of the HIV care cascade in France, published in 2017, although 97% of patients receiving antiretroviral treatment (ART) in the French health system had a viral load below the 200 copies/mL threshold less than 85% still did not reach the first step of the care cascade [4].

Social factors that contribute to HIV-related stigma, as theorised by Goffman, include prejudice, poverty, homophobia, and social and gender inequalities [5]. These pre-existing elements of HIV-related stigma lead to disengagement in care for PLHIV owing to lack of information, negative stigma, and social and economic pressures, all of which contribute to poor health [6, 7]. This results in a change in self-perception after which self-stigmatization emerges with its traditional four dimensions: first, perceived stigma as the perception of how stigmatized groups (here PLHIV) are treated in a given context; second, anticipated stigma as an individual’s worry or anxiety about disclosing their HIV status; third, internalized stigma such as a negative self-image or individuals’ negative feelings about themselves because of HIV; and four, enacted stigma such as others’ discriminatory acts or behaviors towards people with HIV [8, 9]. Overall, PLHIV internalize stigma with the result that the negative attitudes of society and their peers cause them to blame themselves generate low self-esteem or increase existing feelings of shame, guilt, self-blame, fear of dying, fear of hurting or infecting others, fear of being discovered as HIV-positive or homosexual, fear of hurting those around them, and of disappointing, worrying and causing distress to family members [10–12]. As a result, ’protective’ behaviors such as flight, self-exclusion, isolation, withdrawal, the use of deception and denial are mobilized, sometimes leading to aggravating acts against oneself and others, such as party and play (PnP) [13]. On a more positive note, communitarian groupings can lead to activism that restores identity and pride. Gay pride is one example. Ultimately, however, self-stigmatization can become entrenched in the minds of PLHIV. Its negative impact compliance with antiretroviral treatment and on the development of depression is well documented [14]. Individuals adhere to the devaluation of which they are the object and shares the beliefs, representations and opinions that justify the discriminatory measures taken them [15]. Awareness-raising campaigns, targeted or non-targeted information (e.g. U = U), the use of HIV pre-exposure prophylaxis, improvement of the legal and political environment through the promotion and protection of human rights, mobilizing communities, and strengthening people’s self-esteem and social capital were expected to lead to the end of self-stigmatization. Yet, stigma and discrimination against PLHIV both persist [16, 17]. Individual and societal knowledge about HIV/AIDS has not improved or has even worsened, and targeted interventions are still lacking [18, 19]. However, individual resilience factors to self-stigma could be mobilized throughout life, be they individual, familial or extra-familial. A path can be drawn that takes into account movement and mobility within different social spaces and the consistency in the sequence of the actor’s positions along the path, as M. Perez described in men who have sex with men [20]. Certain psychological and psychosocial variables, such as psychological (in)flexibility, mental health, quality of life and recalled parental upbringing, can be predictive of resilience [21–23].

The first aim of our study was to investigate the psychological, social and medical factors that enable resilience to self-stigmatization among PLHIV in France in 2022, assuming that individual factors such as psychological (in)flexibility can be mobilized more readily than social factors, which are slower to develop. Psychological flexibility is the ability to act in a valued direction by accepting the occurrence of difficult psychological events, such as emotions, and unpleasant thoughts. Several studies suggest that psychological flexibility plays a central role in resilience to adverse situations [23, 24]. Hence, our main hypothesis was that greater psychological flexibility would be associated with less HIV self-stigmatization. The second aim of our study was to assess health workers’ perceptions of internalized stigma in their patients living with HIV, and the mobilizing care they provide.

## Methods

We conducted a multicenter observational study in France from 15 January to 15 June 2022 involving two clinicians who agreed to participate through regional coordinated care organizations (COREVIH), and PLHIV receiving care from these outpatient clinic doctors.

### Conduct of the survey

Before the survey, the research center sent a letter describing the study to the 28 presidents of COREVIH centers in France and the overseas departments. They in turn passed on the information to the HIV doctors in the local hospitals. If one of these doctors accepted to take part in our study, he or she would send us an enrolment form and then receive all the study documents, including the surveys.

After reading the information letter during the study month, doctors at the participating centers completed (once) the anonymized paper doctor survey and asked all PLHIV they had seen in consultation to complete (once) the anonymized paper patient survey. At the end of the consultation, PLHIV completed the survey in French only, and could be helped if they did not read and/or speak French. Patients could request to see a psychologist during or after the survey if required.

### Sampling

Inclusion criteria for clinicians were those caring for PLHIV who agreed to participate. The exclusion criterion was refusal to participate.

Inclusion criteria for PLHIV were individuals older than 18 years who had been on ART for more than six months. The exclusion criterion was refusal to participate.

### Data collected

Demographic variables collected from PLVIH participants included gender, age, geographic origin, precariousness, drug use, sexuality, family context, employment, leisure time activities, and care setting. Medical explanatory variables were duration of HIV infection, duration of antiretroviral treatment, HIV plasma viral load, addictions, comorbidities, and care pathways. Self-stigmatization was measured by a Canadian questionnaire that explored four domains: (i) perceived stigma as the perceived consequence of other people knowing about an individual’s HIV status, (ii) anticipated stigma as an individual’s concern or worry about disclosing their HIV status, (iii) internalized stigma such as a negative self-image or negative feelings individuals have about themselves because of HIV, and (iv) enacted stigma such as discriminatory acts or behaviors by people living with HIV [8, 9]. These four aspects of stigmatization were assessed using scales ranging from 1 to 4 (from strongly disagree to strongly agree).

The other variables measured were: (i) quality of life [25], (ii) anxiety and depression [26], (iii) psychological flexibility, as assessed by the Multidimensional Psychological Flexibility Inventory short form (MPFI-24) [27], (iv) inflexibility, generated by the avoidance of internal events such as thoughts, emotions and sensations, (v) cognitive fusion (the tendency to be attached to our psychological events and to take our thoughts as real facts) [28], (vi) social provision [29], and (vii) perception of parental support, derived from Parental Educational Practices Perceived by the Child (PEPPE) [30].

The questionnaire for clinicians explored the perception of self-stigmatization among the people they cadre for and the means used to combat self-stigmatization. Some demographic variables were collected including age, gender, profession and type of practice, location and geographical area of work, specialty and training, and seniority in caring for PLHIV. Standardized questions on self-stigmatization were adapted from those asked of PLHIV.

#### Regulatory considerations

This observational study (Clinical Trials N° 2021-A01588-33/SI:21.02814.000036) was pre-designed to comply with the French MR003 research standard (health research without obtaining consent) and was reviewed and approved by the CPP Marseille Ethics Committee on November 23, 2021 under the internal reference number 221 C37MS01. Verbal consent was obtained, confirmed and documented by the patient’s completion of the paper self-questionnaire.

To ensure data security and safe storage, both patients and clinicians completed an anonymized paper copy of the questionnaire, which was sent immediately upon completion to the clinical trial technician, who generated their anonymized personal data as an electronic case report form (eCRF) using a REDCap web application developed specifically for this study [31]. Access to the data was restricted to the clinical research center (Clermont-Ferrand University Hospital), which was responsible for data monitoring, security, privacy and control.

#### Statistical analysis

Sample size was estimated on the basis of on a previous study [20], which found significant positive correlations between psychological distress and self-stigmatization (r = 0.33 and r = 0.41) depending on the self-stigmatization variables considered. Thus, for a correlation coefficient of approximately 0.30 (the case requiring the largest number of subjects), 112 subjects would be required to reject the hypothesis that the coefficient is null, with a two-sided alpha level of 5% and a power of 90%. However, this number of subjects would not be sufficient to estimate the prevalence of self-stigmatization among PLHIV. For an expected rate of self-stigmatization of about 50% (the case requiring the largest number of subjects), the inclusion of at least 600 PLHIV allowed for a rate precision of ±4%. A total of 600 PLHIV were planned to be included.

Statistical analyses were performed with Stata software (version 15, StataCorp, College Station, Texas, USA) and Jamovi (version 2.3.28.0). All tests were two-tailed with an alpha level of 5%. Categorical variables are expressed as number of subjects and corresponding percentages, and continuous variables as mean ± standard deviation or median [25^th^, 75th percentiles] according to their statistical distribution. The proportion of self-stigmatization (past and/or present) is presented with a 95% confidence interval (95% CI). Factors associated with self-stigmatization (past and/or present) were examined with Chi-squared test or Fisher’s exact test for categorical variables and Student’s t-test or Mann-Whitney test for continuous variables. Multivariate analysis (multiple logistic regression) was then performed, taking into account covariates in the light of univariate results and clinical relevance. Results are expressed as odds ratios (ORs) and 95% CIs. Factors associated with each of the four domains of self-stigmatization were examined using the Mann-Whitney test for binary categorical variables, the Kruskal-Wallis test for categorical variables with more than two categories, and Spearman’s correlation coefficient for continuous variables. Multivariate analyses were then performed (multiple regressions) and results are expressed as regression coefficients and 95% CIs.

## Results

The survey was conducted in 45 care services across France and completed by 131 doctors who had seen 666 PLHIV who had attended consultations during the study period and agreed to participate in the survey.

### Descriptive characteristics of the PLHIV sample

Descriptive statistics on the socio-demographic, psychological and medical characteristics of the respondents are presented in Table 1.

**Table 1:** Demographic, psychosocial and medical characteristics of PLHIV responders.

		n (%) mean±standard deviation median [25th; 75th percentiles]
**Age** ^**1**^	Years	53.5±2.6
**Gender** ^**2**^	Man	471 (71.0%)
	Woman	188 (28.4%)
	Transgender	4 (0.6%)
**Lifestyle** ^**3**^	With a partner	327 (49.4%)
**Sexual orientation** ^**4**^	Heterosexual	295 (45.0%)
	Homosexual	286 (43.7%)
	Other / declined to respond	74 (11.3%)
**Children** ^**5**^	One or more	261 (40.8%)
**Country of birth** ^**6**^	France	497 (75.4%)
	Sub-Saharan Africa	82 (12.4%)
	Other	80 (12.1%)
**Educational level** ^**7**^	Secondary education completed	385 (58.7%)
**Professional activity** ^**6**^	Employment / training	351 (53.3%)
	Retired	146 (22.1%)
	Other	162 (24.6%)
**Housing** ^**8**^	At home	592 (90.9%)
	Sheltered	57 (8.8%)
	Homeless	2 (0.3%)
**Precariousness** ^**9**^	EPICES score	30.8 [16.6; 47.9]
	Precarious (EPICES score ≥30.17)	313 (54.1%)
**Location-based dating sites** ^**1**^	Use	127 (19.1%)
**HAD scale**	Anxiety scale^10^	7 [5; 11]
	Anxiety ^10^ ≤7	342 (53.5%)
	Depression scale^11^	4 [2; 7]
	Depression^11^ ≤7	502 (78.1%)
**Psychological Flexibility scale (MPFI)**	Flexibility^12^	4.4 [3.8; 5.1]
	Inflexibility^13^	3.0 [2.5; 3.8]
**Social provision scale**	Social provisions ^14^	78 [70; 86]
**Parental support in childhood** ^**15**^	Good	336 (53.2%)
	Not so good	218 (34.5%)
	Bad	77 (12.2%)
**Quality of life**	Very good or good^16^	452 (69.2%)
	Poor very poor	168 (25.7%)
	Neither good nor poor	33 (5.1%)
	Satisfied or very satisfied with their health^7^	393 (59.9%)
	Not satisfied or not at all satisfied	169 (25.8%)
	Neither satisfied nor unsatisfied	94 (14.3%)
**Last HIV viral load** ^**17**^	Undetectable	601 (90.9%)
	Detectable	40 (6.1%)
	Do not know	20 (3.0%)
**Time to VIH detection** ^**18**^	Years	19 [8; 28]
**Duration of ARV treatment** ^**19**^	Years	15 [7; 24]
**Smoking** ^**17**^	Active	209 (31.6%)
	Ex-smoker	105 (15.9%)
**Alcohol consumption**	Once a week or more	315 (48.2%)
**Illicit drug use** ^**17**^	Active	81 (12.6%)
	Past	69 (10.7%)
**Lipodystrophy** ^**20**^	Presence	141 (21.7%)
	Absence	435 (67.0%)
	Do not know	73 (11.3%)
**Important body** **Modification** ^**19**^	Absence	590 (90.0%)
	Presence	66 (10%)
**Loss of intellectual capacity** ^**21**^	Yes	91 (13.9%)
**Associated treatment** ^**22**^	Yes	350 (53.1%)
	Anti-hypertensive treatment	147 (42%)
	Psychiatric targets	104 (29.7%)
	Lipid lowering	81 (23.1%)
	Cardiovascular targets	53 (15.1%)
	To counter loss of desire	41 (11.7%)
	Antidiabetic	37 (10.6%)
	Osteoarticular targets	37 (10.6%)
	Neurological targets	29 (8.3%)
	Renal targets	25 (7.1%)
	For addiction withdrawal	16 (4.6%)
	Anticancer	11 (3.1%)
	Anti-hepatitis B or C	10 (2.8%)
	Unspecified	39 (11.1%)
**HIV follow-up** ^**23**^	In university hospital	375 (58.9%)
**Consultations within the year**	Once or more with primary care physician ^24^	482 (77.0%)
	Once or twice with HIV specialist^2^^5^	489 (74.4%)
	Once or more with other specialists^17^	455 (69.6%)

PLHIV: people living with HIV

HAD: Hospital Anxiety and Depression

ARV: antiretroviral treatment

^1^ 666 responders

^2^ 663 responders

^3^ 662 responders

^4^ 655 responders

^5^ 640 responders

^6^ 659 responders

^7^ 656 responders

^8^ 651 responders

^9^ 579 responders

^10^ 639 responders

^11^ 643 responders

^12^ 574 responders

^13^ 568 responders

^14^ 515 responders

^15^361 responders

^16^ 653 responders

^17^ 661 responders

^18^ 619 responders

^19^ 556 responders

^20^ 654 responders

^21^ 649 responders

^22^ 657 responders

^23^ 659 responders

^24^ 637 responders

^25^ 626 responders

#### Descriptive characteristics of the sample of health workers

Table 2 presents descriptive statistics of the socio-demographic characteristics of the 131 health workers surveyed.

**Table 2:** Characteristics of the 131 health workers.

		n (%) mean±standard deviation median [25th; 75th percentiles]
**Occupation**	Doctor	95 (72.5%)
	Nurse	26 (19.9%)
	Psychologist	5 (3.8%)
	Other	5 (3.8%)
**Age**	Years	45.1±10.8
**Gender**	Man	42 (32.1%)
	Woman	87 (66.4%)
	Missing data	2 (1.5%)
**Specialty**	Infectious diseases	83 (63.4%)
	General practice	17 (13.0%)
	Other	15 (11.5%)
	Missing data	16 (12.2%)
**Practice mode**	Full-time	106 (80.9%)
	Part-time	25 (19.1%)
**Seniority in the care of PLHIV**	Years	11 [6; 25]
**Place of work**	University hospital	62 (47.3%)
	General hospital	63 (48.1%)
	**City exercise**	5 (3.8%)
	Missing data	1 (0.8)
**Geographic area**	Urban	114 (87.0%)
	Rural	14 (10.7%)
	Missing data	3 (2.3%)
**Trainings**	Addictology	35 (26.7%)
	HIV psychology	45 (34.4%)
	Sexual health	67 (51.1%)
	Migrant health	44 (33.6%)

PLHIV: people living with HIV

#### Self-stigmatization experienced by PLHIV

Having read the definition of self-stigmatization as the process through which environmental stigmas impact on self-image, PLHIV responded to the question of whether their self-stigmatization was present and/or past: 387 (58.1%) of the 666 PLHIV reported no past or present self-stigmatization, 87 (13.1%) past, 148 (22.2%) past and present, and 44 (6.6%) present self-stigmatization (Table 3). Thus, 41.9% (95% CI: 38.1 to 45.7%) of PLHIV reported past and/or present self-stigmatization. Those who experienced self-stigmatization always perceived a decrease when they were with their family or a partner, at work, during leisure time or when playing sports, but an increase when confronted with themselves. The self-stigmatization issues identified by 223 respondents were difficulties in sexual and affective relationships rather than social relationships. Few participants felt that the impact of COVID-19 was negative.

**Table 3:** Self-stigmatization experienced by PLHIV.

			N (%) median [25^th^; 75^th^ percentiles]
**Presence of self- stigmatization** ^**1**^	Never		387 (58.1%)
	Before		87 (13.1%)
	Now		44 (6.6%)
	Before and now		148 (22.2%)
**Circumstances of self-stigmatization** ^**1**^	At work	Previously	108 (16.2%)
		Now	65 (9.8%)
	At leisure	Previously	89 (13.4%)
		Now	70 (10.5%)
	At home	Previously	108 (16.2%)
		Now	76 (11.4%)
	With a partner	Previously	91 (13.7%)
		Now	51 (7.7%)
	Sports activities	Previously	59 (8.9%)
		Now	46 (6.9%)
	Job search	Previously	50 (7.5%)
		Now	31 (4.7%)
	Face to face	Previously	97 (14.6%)
		Now	108 (16.2%)
	Other	Previously	14 (2.1%)
		Now	15 (2.3%)
**Consequences of self-stigmatization** ^**2**^	Isolation		99 (44.4%)
	Difficulties with emotional and sexual life		137 (61.4%)
	Social difficulties		97 (43.5%)
	Other		15 (6.7%)
**Self-deprecation** ^**3**^	Always		18 (2.7%)
	Sometimes		132 (20.2%)
	Not anymore		95 (14.5%)
	Never		410 (62.6%)
**Causes of self-deprecation** ^**4**^	Physical characteristics		30 (21.3%)
	Skin color		6 (4.3%)
	Sexual orientation		8 (5.7%)
	Social status		21 (14.9%)
	Financial resources		30 (21.3%)
	Paid sex		4 (2.8%)
	Addictions		6 (4.3%)
	HIV infection		95 (67.4%)
	Age		28 (19.9%)
	Diminished capacity		23 (16.3%)
	Other		6 (4.3%)
**Disclosing HIV status to health workers** ^**5**^	To all		425 (64.5%)
	To most		173 (26.2%)
	Never		61 (6.3%)
**Effect of COVID-19 on stigma** ^**6**^	None		513 (81.2%)
	Deterioration		77 (12.2%)
	Improvement		28 (4.4%)
	Unspecified effect		14 (2.2%)
**Stigma scales**	Perceived stigma^7^		1.46 [1.15; 2.23]
	Anticipated stigma^8^		2.89 [2.33; 3.33]
	Internalized stigma^9^		1.80 [1.50; 2.20]
	Enacted stigma^10^		1.94 [1.63; 2.31]

PLHIV: people living with HIV

^1^ 666 responders

^2^ 223 responders

^3^ 655 responders

^4^ 141 responders

^5^ 659 responders

^6^ 632 responders

^7^ 466 responders

^8^ 488 responders

^9^ 482 responders

^10^ 434 responders

^11^ 639 responders

^12^ 643 responders

^13^ 574 responders

^14^ 568 responders

^15^ 515 responders

Overall, 425/659 (64.5%) disclosed their HIV status to all health care providers, 150/655 (22.9%) reported occasional or severe self-deprecation, and most (67.4%) of the 141 respondents cited HIV infection as a reason for self-deprecation.

The median response of 448 PLHIV to anticipated stigma indicated agreement (2.89 [2.33; 3.33]). In addition, the median responses of 482 respondents to questions about internalized stigmatization tended towards disagreement (1.80 [1.50; 2.20]), the median responses of 466 respondents to questions about perceived stigma tended towards stronger disagreement (1.46 [1.15; 2.23]), and the median responses of 434 respondents to questions about enacted stigma tended towards moderate disagreement (1.94 [1.63; 2.31]).

#### Factors associated with self-stigmatization among PLHIV

In univariate analysis, the PLHIV characteristics associated with self-stigmatization (past and/or present) were (i) demographic characteristics such as younger age (p = 0.03), not living with a partner (p = 0.04), not having a job (p = 0.001), and precariousness (p = 0.005), (ii) presence of addictions: smoking (p = 0. 002), drug use (p = 0.03), (iii) presence of therapeutic side effects: lipoatrophy (p = 0.03), major physical changes (p<0.001), (iv) comorbidities and care pathways: loss of intellectual capacity (p = 0.02), being treated for anxiety-depressive syndrome (p<0. 001), not being treated for hypertension (p = 0.009), more than two HIV consultations in the last year (p = 0.04), (v) and psychosocial characteristics: negative COVID-19 pandemic effect on self-stigmatization (p<0.001), self-deprecation (p<0.001), not having a good or very good quality of life (p<0. 001), not being satisfied or very satisfied with one’s health (p<0.001), anxiety (p<0.001), depression (p<0.001), low psychological flexibility (p = 0.001), inflexibility (p<0.001), reduced social provision (p<0.001), and lack of parental support in childhood (p<0.001).

**Fig 1:**
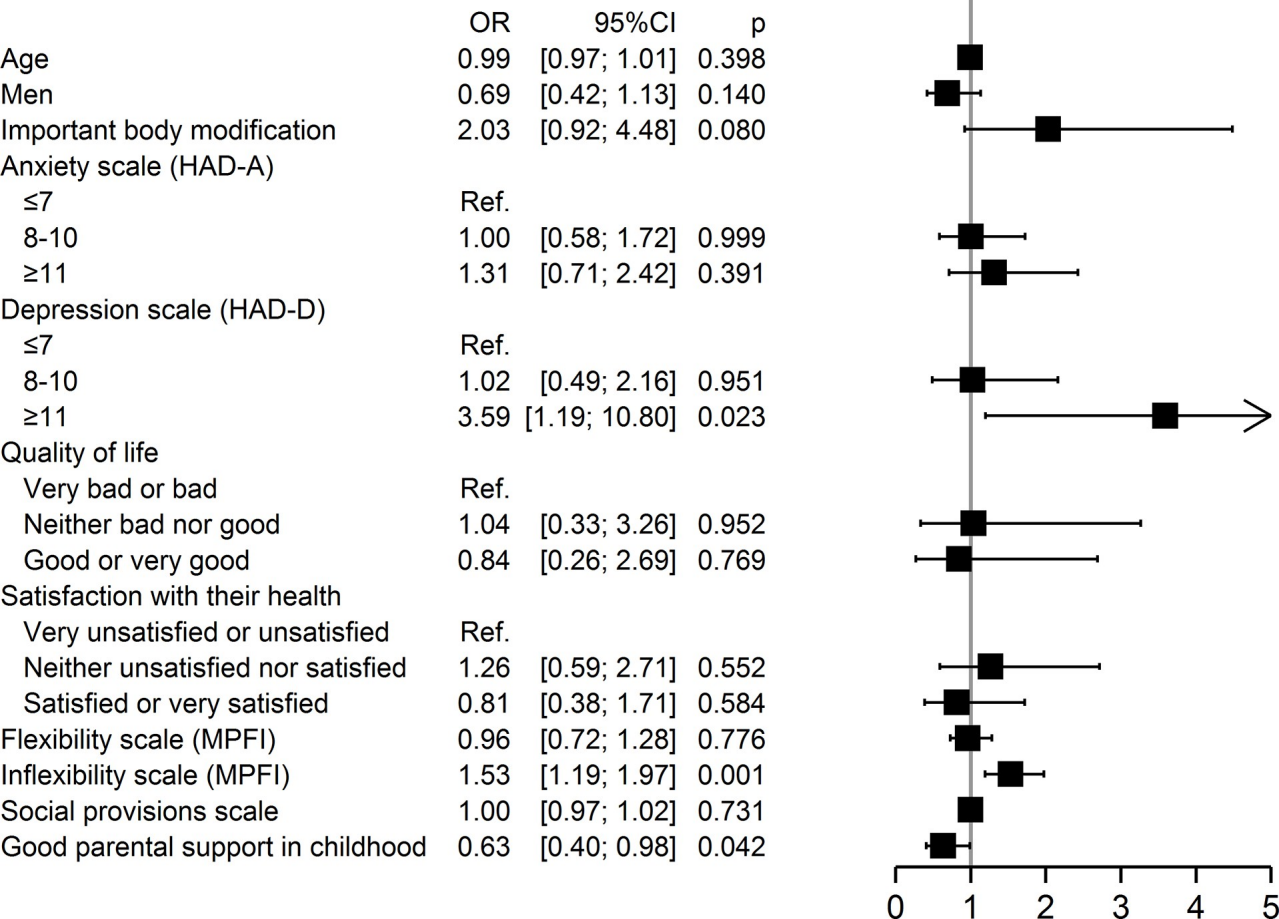
Multivariate analysis of factors associated with present and/or past self-stigmatization (n = 424).

Multivariable analysis showed (Fig 1) that major depression (OR: 3.59, 95% CI: 1.19 to 10.80, p = 0.02), inflexibility (OR: 1.53, 95% CI: 1.19 to 1.97, p = 0.001), and lack of parental support in childhood (OR parental support: 0.63, 95% CI: 0.40 to 0.98, p = 0.04) were associated with self-stigmatization.

### Components of (in)flexibility associated with (more) less self-stigmatization among PLHIV

Among the dimensions of psychological flexibility most negatively related to HIV self-stigmatization, only cognitive defusion (the ability to distance oneself from unwanted experiences without getting caught up in them) was significantly (negatively) related to HIV stigma when other components were statistically controlled (Table 4). Among the dimensions of psychological inflexibility most positively related to HIV self-stigmatization, only cognitive fusion (getting caught up in unwanted internal experiences) was positively and significantly related to HIV stigma when other components were statistically controlled (Table 5). Finally, when both defusion and fusion were entered simultaneously in a multiple regression analysis, both remained significantly related to HIV self-stigmatization in the expected direction (β = -0.16, p < 0.01; β = 0.28, p < 0.001, respectively).

**Table 4:** Relationships between psychological flexibility components and HIV self-stigmatization.

	Zero-order *r*	Standardized beta coefficient
*Flexibility components*		
Acceptance	0.00	-
Contact with the present moment	-0.03	-
Self as context	-0.11*	0.08
Defusion	-0.26***	-0.28***
Values	-0.16**	-0.02
Committed action	-0.16**	-0.04

*Note*: The zero-order column indicates the variable zero-ordered effects, with other variables not being statistically controlled for in the correlation analysis. The standardized beta coefficient gives the effect of each variable when other variables are statistically controlled for. * p < 0.05, ** p < 0.01, *** p < 0.001.

**Table 5:** Relationships between psychological inflexibility components and HIV self-stigmatization.

	Zero-order *r*	Standardized beta coefficient
*Inflexibility components*:		
Experiential avoidance	-0.08	-
Lack of contact with present moment	0.06	-
Self as content	0.17**	-0.03
Fusion	0.34***	0.23**
Lack of contact with values	0.31***	0.14
Inaction	0.30***	0.05

*Note*: The zero-order column indicates the variable zero-ordered effects, with other variables not being statistically controlled for in the correlation analysis. The standardized beta coefficient gives the effect of each variable when other variables are statistically controlled for.

* p < 0.05

** p < 0.01

*** p < 0.001.

#### Factors associated with each domain of self-stigmatization among PLHIV

**Fig 2:**
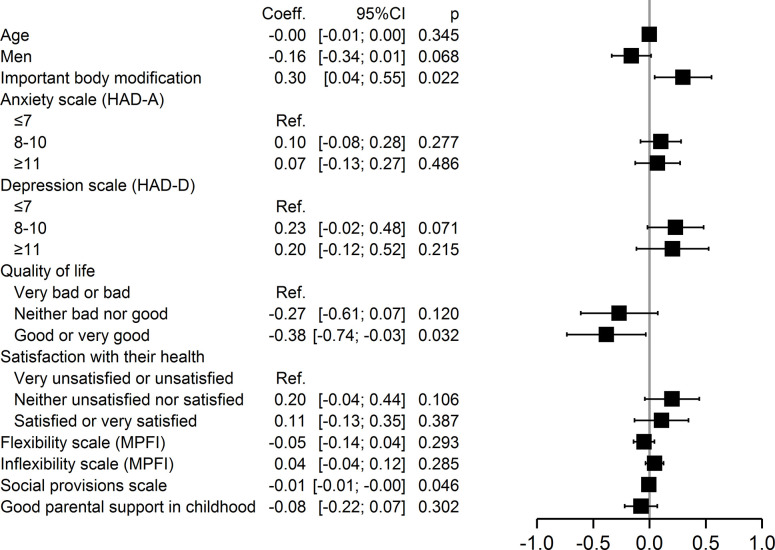
Multivariate analysis of factors associated with perceived stigmatization.

**Fig 3:**
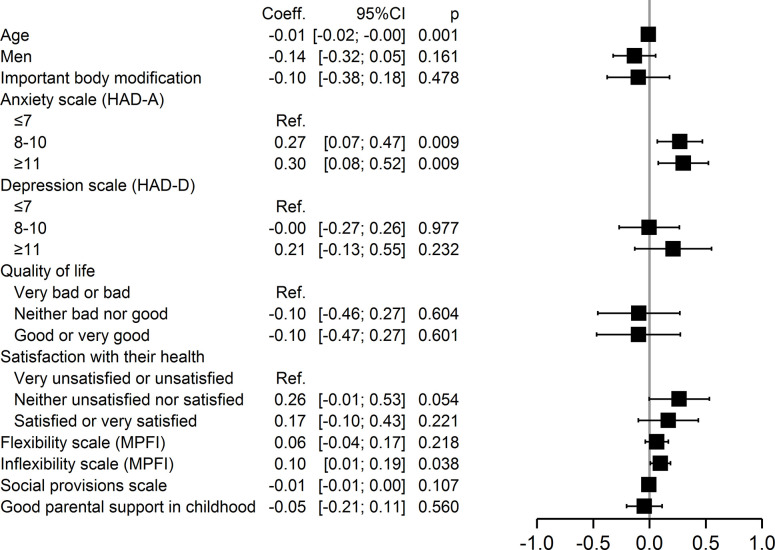
Multivariate analysis of factors associated with anticipated stigmatization.

**Fig 4:**
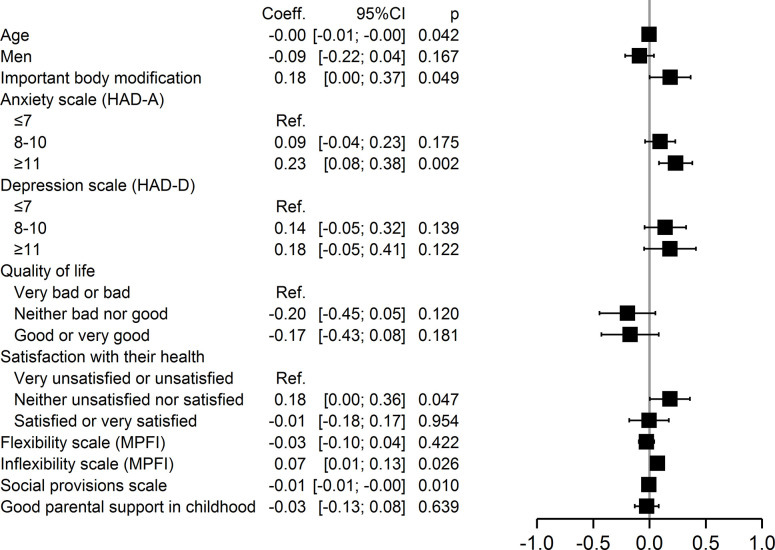
Multivariate analysis of factors associated with internalized stigmatization.

**Fig 5:**
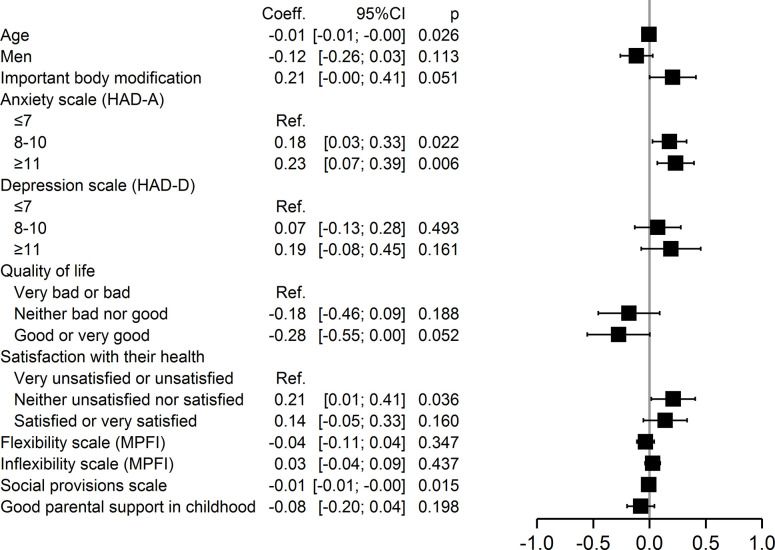
Multivariate analysis of factors associated with enacted stigmatization.

In addition, multivariable analysis also identified specific factors within each of the four domains of self-stigmatization. Major physical changes (p = 0.02) were associated with perceived stigma, whereas good or very good quality of life (p = 0.03) and good social provision (p = 0.046) were protective (Fig 2). Anxiety (p = 0.009) and inflexibility (p = 0.04) were associated with anticipated stigma, but younger age (p = 0.001) was protective (Fig 3). Severe anxiety (p = 0.002), being neither satisfied nor dissatisfied with one’s health (p = 0.047), and inflexibility (p = 0.026) were associated with internalized stigma, but younger age (0.042) and good social provision (0.01) were protective (Fig 4). Moderate (p = 0.02) and severe anxiety (p = 0.006) and being neither satisfied nor dissatisfied with one’s health (p = 0.04) were associated with enacted stigma, whereas younger age (p = 0.026) and social provision were protective (p = 0.015) (Fig 5).

### Health worker perceptions of self- stigmatization of their patients living with HIV

The prevalence of self-stigmatization was estimated to be low by more than half of the health workers surveyed (Table 6). A total of 69.2% estimated that HIV infection is associated with self-stigmatization, 56.7% that sexual orientation is associated with self-stigmatization, and 52.9% that social conditions are associated with self-stigmatization. The most important circumstances leading to self-stigmatization, according to health workers, were family and work. Many health workers associated self-stigmatization with poor contact, depression and/or poor perception of health. For health workers, the COVID-19 pandemic had increased the feeling of stigmatization for half of PLHIV.

**Table 6:** Health worker perceptions of self-stigmatization of their patients living with HIV.

		n (%)
**Estimated prevalence of PLHIV self-stigmatization**	None	27 (20.6%)
	Low	72 (55.0%)
	Medium	31 (23.7%)
	Strong	1 (0.8%)
**Opinions on the causes of PLHIV self- stigmatization**	Physical characteristics	14/104 (13.5%)
	Skin color	22/104 (21.2%)
	Sexual orientation	59/104 (56.7%)
	Social status	55/104 (52.9%)
	Financial resources	26/104 (25.0%)
	Paid sex	9/104 (8.7%)
	Addictions	30/104 (28.8%)
	HIV infection	72/104 (69.2%)
	Other	12/104 (11.5%)
**Opinions on circumstances of PLHIV self-stigm**a**tization**	At work	85/110 (77.3%)
	At leisure	71/110 (64.5%)
	At home	91/110 (82.7%)
	Living with a partner	59/110 (53.6%)
	Sports activities	38/110 (34.5%)
	Job search	62/110 (56.4%)
	Face to face	42/110 (38.2%)
	Other	7/110 (6.4%)
**Effect of COVID-19 on PLHIV self- stigm**a**tization**	Deterioration	57 (43.5%)
	Improvement	1 (0.8%)
	No effect	63 (48.1%)
	Missing data	10 (7.6%)
**Assessment links of health workers to PLHIV self-stigmatization**	Uncontrolled viral load	13 (9.9%)
	Lack of compliance	33 (25.2%)
	Depression	91 (69.5%)
	Suicidal ideation	31 (23.7%)
	Poor health perception	88 (67.2%)
	Fewer social contacts	99 (75.6%)
	Lack of family attachment	27 (20.6%)
	Distinctive psychology	0 (45.8%)
	Changes in self-image	64 (48.9%)
	Age	15 (11.5%)
	Recurring problems	46 (35.1%)
	Missing data	3 (2.3%)
**Management of PLHIV self-stigmatization**	Like the others	15 (11.5%)
	Psychiatric care	39 (29.8%)
	Therapeutic education	41 (31.3%)
	Associated social care	27 (20.6%)
	Psychological support	85 (64.9%)
	Multidisciplinary discussion	31 (23.7%)

PLHIV: people living with HIV

The methods of care proposed by the health workers surveyed were frequent psychological support (64.9%), combined with therapeutic patient education (33%), psychiatric consultation and, to a lesser extent, social support (20.6%).

We evaluated the characteristics of caregiver-associated identification and awareness of self-stigmatization, but found no significant difference between their characteristics and those of others.

## Discussion

The 666 PLHIV recruited, of whom 471 (71%) were men, had a median age of 53.5±2.6 years, and had been receiving ART for a median of 15 years [7; 24]. Of the total number, 601 (90.9%) had an undetectable viral load, 350 (53.1%) had one or more treated comorbidities, and 435 (67.0%) self-reported no lipodystrophy. Overall, 42% had experienced current and/or past self-stigmatization, a rate that was underestimated by the majority of healthcare professionals, of whom only 21% accurately estimated the prevalence of self-stigmatization among the PLVIH. We found evidence that important factors associated with self-stigmatization were: i) the protective factor of parental support during childhood, ii) psychological inflexibility and, in particular, cognitive fusion, i.e. the tendency to become attached to our psychological events (thoughts, emotions), to take them as reality and to be influenced by them in our behavior, all of which interrupt contact with the present moment, and iii) major depression. Age, gender and country of birth were not found to be associated with self-stigmatization among PLVIH in our study.

These findings concur partly with those of a review of 176 studies that found consistent associations between self-stigmatization and negative psychological variables (e.g. depression and anxiety), social variables (e.g. lack of social support, discrimination, non-disclosure and intersecting stigmas), and health variables (e.g. substance use, non-adherence, and negative clinical HIV outcomes) [32]. The authors of the review argued for a socio-ecological approach to self-stigmatization. We agree, but emphasize the need to mobilize individual factors while waiting for societal change. For people with depression and/or anxiety, psychiatric treatment needs to be available. For people with psychological traits such as psychological inflexibility, we advocate the use of psychological reinforcement in association with acceptance and commitment therapy, a representative approach of the third wave of cognitive-behavioral therapies that can be applied to PLHIV [33]. This approach help people resolve feelings of being stuck, cope with stress, improve their wellbeing and build a more meaningful life around what they really value. If necessary, depression and self-esteem therapies can be offered.

While some physical changes are unavoidable, such as those associated with ageing, others, such as those induced by antiretroviral treatment, have a negative impact on perceived stigma. Clinicians need to be vigilant, as some therapy-related physical changes can be prevented [34]. In addition, they should not forget to apply for the assistance of a social worker and social welfare support, when necessary.

However, individual support will certainly not suffice and much remains to be done, not only at the institutional and policy level. According to the work of Nenghem Mensah and Ken Monteith, self-stigmatization is an anticipated and/or internalized stigma that cannot be separated from institutional stigmatization resulting from the application of policies, laws, and procedures that may exist in different settings such as prisons, justice systems, police, financial institutions, and certain religious institutions, or from concrete interpersonal and community stigmatization that occurs through overt acts of discrimination such as exclusion, physical or psychological abuse, and bullying [35]. Not all health workers interviewed in our study recognized self-stigmatization among PLHIV, and their perceptions of self-stigmatization are very difficult to grasp. Analysis of the condition should be based on a complex reading. The Canadian scale used in our study has not yet been fully adopted by French health professionals and health workers therefore rely on their own criteria to assess self-stigmatization, which probably makes our study variables inappropriate for identifying factors associated with perceptions of self-stigmatization. Overall, 65% of health workers nevertheless reported that they provide psychological support to PLHIV who exhibit self-stigma. However, the fact remains that these workers do not diagnose or assess self-stigmatization in their patients and hence likely underestimate it.

Among the causes of self-stigmatization, health workers expected to observe intersectional stigmatization, such as physical characteristics, ethnic and/or cultural origin, substance use, sexual orientation, mental disorders, gender identity, sex work, language, social class, and HIV, whereas the PLHIV in their majority spoke of HIV and very few of sexual orientation. Is this a specifically French tendency? Or does it reflect a bias on the part of respondents who were interviewed within their HIV care structure and therefore had HIV at the forefront of their preoccupations? If the circumstances in which self-stigmatization is most likely to occur are family and work, and are mentioned as such by both health workers and PLHIV, the space in which self-stigmatization is now most likely to be reported is in face-to-face encounters. This realization should encourage health workers to provide PLHIV with specific psychological care.

We would also like to shed more light on anticipated stigma, one of the four aspects of self-stigmatization. In our survey, 50% of the PLHIV responded positively to questions about individual concerns or worries about disclosing their HIV status. However, only 29% of participants were currently experiencing self-stigmatization. These findings support the opinion of Daskalopoulou that the decision not to disclose may be a means of coping and is not necessarily associated with negative psychological consequences or difficulties in managing treatment [36].

Indeed, and this is the main strength of our study, there seems to be an urgent need to reduce self-stigmatization, which is reported as current experience in 28.8% of PLHIV surveyed in France and as current or past experience in 42%. Self-stigmatization can occur at any time in a person’s life and should be addressed by doctors as soon as the patient is diagnosed with HIV. Measures could be taken to inform health workers about the challenges faced by PLHIV and to screen PLHIV for self-stigmatization using reliable tools in order to provide appropriate therapy.

Our study has certain limitations. We tried to minimize recruitment bias by consecutively recruiting all PLHIV who agreed to participate and by strictly defining the criteria for inclusion and exclusion of patients. PLHIV who did not read French were able to be included in the study because health workers could help them, but this was not the case for those who did not speak the language. Thus, although patients and doctors were enrolled voluntarily, the characteristics of PLHIV in the study are the same as those of PLHIV in the French Hospital Database on HIV Infection (FHDH). This assertion is based on the following variables: age, percentage of patients with an undetectable viral load, duration of antiretroviral treatment, and comorbidities, in particular anxiety-depressive syndromes. Our sample can therefore be considered as largely representative of the French PLHIV population [37, 38]. The French Vespa study also provides information on the number of HIV-positive people with anxiety-depressive syndrome, and, like our study, reports a link with self-stigmatization [39].

Finally, in line with our findings, an Italian study that aimed to assess the impact of HIV-related self-stigmatization on care engagement and mental health emphasized the importance of supporting PLHIV to find strategies to cope with stigma-related stress and optimize care engagement [40].

## Conclusion

Resilience factors can be mobilized and so there is an urgent need to assess current self-stigmatization, which affects one third of PLHIV. Once self-stigmatization is well mapped, one way to address it might be to combine therapies for depression and sequelae associated with lack of parental support with new cognitive behavioral therapies. Dealing with inflexibility requires appropriate care, and balance between therapeutic approaches. In addition, clinicians should be cautious and only prescribe antiretroviral treatments that induce no physical changes. They should also explain the basics of nutrition and the importance of physical exercise in preventing weight gain and propose social support when necessary. At the same time, we must continue to combat the interpersonal, community and institutional stigmas that trigger self-stigmatization.
